# The forgotten key players in rheumatoid arthritis: IL-8 and IL-17 – Unmet needs and therapeutic perspectives

**DOI:** 10.3389/fmed.2023.956127

**Published:** 2023-03-22

**Authors:** Elisa Gremese, Barbara Tolusso, Dario Bruno, Simone Perniola, Gianfranco Ferraccioli, Stefano Alivernini

**Affiliations:** ^1^Division of Clinical Immunology, Fondazione Policlinico Universitario A. Gemelli-IRCCS, Rome, Italy; ^2^Immunology Core Facility, Fondazione Policlinico Universitario A. Gemelli-IRCCS, Rome, Italy; ^3^School of Medicine, Università Cattolica del Sacro Cuore, Rome, Italy; ^4^Department of Medicine, University of Verona, Verona, Italy; ^5^Division of Rheumatology, Fondazione Policlinico Universitario A. Gemelli-IRCCS, Rome, Italy

**Keywords:** interleukin-8, interleukin-17, rheumatoid arthritis, organ damage, chronic pain

## Abstract

Despite the relevant advances in our understanding of the pathogenetic mechanisms regulating inflammation in rheumatoid arthritis (RA) and the development of effective therapeutics, to date, there is still a proportion of patients with RA who do not respond to treatment and end up progressing toward the development of joint damage, extra-articular complications, and disability. This is mainly due to the inter-individual heterogeneity of the molecular and cellular taxonomy of the synovial membrane, which represents the target tissue of RA inflammation. Tumor necrosis factor alpha (TNFα) and interleukin-6 (IL-6) are crucial key players in RA pathogenesis fueling the inflammatory cascade, as supported by experimental evidence derived from *in vivo* animal models and the effectiveness of biologic-Disease Modifying Anti-Rheumatic Drugs (b-DMARDs) in patients with RA. However, additional inflammatory soluble mediators such as IL-8 and IL-17 exert their pathogenetic actions promoting the detrimental activation of immune and stromal cells in RA synovial membrane, tendons, and extra-articular sites, as well as blood vessels and lungs, causing extra-articular complications, which might be excluded by the action of anti-TNFα and anti-IL6R targeted therapies. In this narrative review, we will discuss the role of IL-8 and IL-17 in promoting inflammation in multiple biological compartments (i.e., synovial membrane, blood vessels, and lung, respectively) in animal models of arthritis and patients with RA and how their selective targeting could improve the management of treatment resistance in patients.

## Introduction

### The synovial membrane is a key tissue in rheumatoid arthritis

The synovial membrane (SM), which covers the joints and lines the joint cavity, is made up of two layers: lining and sublining (1–3). The *lining layer* is made up of two/three cell layers and does not have a basement membrane. The cell types of the lining layer are resident synovial macrophages and fibroblast-like synoviocytes (FLS) that reside over an underlying connective tissue. This underlying connective tissue is the sublining layer that is characterized by sparse tissue-resident macrophages and fibroblasts, adipose cells, and blood and lymphatic vessels as well as minimal infiltrating inflammatory cells. In the last few years, the use of high-definition technologies such as single-cell RNA sequencing revealed the heterogeneity of these populations and novel cell–specific functions (4–6). In particular, in healthy conditions, synovial tissue-resident macrophages and fibroblasts play a crucial homeostatic function forming an immunological barrier that enables the isolation of the joint cavity through their expression of membrane tight junctions and by the release of regulatory and anti-inflammatory mediators maintaining joint homeostasis (4–7). Few mast cells (MCs) have been described in normal synovium, mainly the subset containing either tryptase (MCt) or chymase (MCtc) (8). In the collagen-induced arthritis (CIA) model, MCs emerged as players of the early immune-driven arthritis (not in the effector phase of the immune response in the K/BxN model) (9), and MCt is thought to contribute to the tissue hyperplasia by inhibiting FLS apoptosis (10). During arthritis development, immune cells are recruited toward the synovial membrane that shows lining layer hyperplasia and becomes a tumor-like tissue (pannus). The pannus formation presents a molecular basis and pathways leading synovial fibroblasts to achieve and maintain an aggressive phenotype, similar to locally invasive cancer (3). Analyzing the physiology of FLS, it was demonstrated that normal FLS and RA-FLS spontaneously express genes such as IL-6, IL-8 (CXCL8), and transforming growth factor ß1 (TGFß1) but not tumor necrosis factor alpha (TNFα). After LPS stimulation, normal FLS and RA-FLS expressed granulocyte-macrophage colony-stimulating factor (GM-CSF) and interleukin-1α (IL-1α) (11). Interestingly, another study showed that long-standing RA-derived FLS constitutively expressed basic fibroblast growth factor (FGF), TGFß, IL-1α, and IL-6 but not TNFα (12), suggesting that FLS are constitutively prone to synthesize IL1-α and ß and mostly IL-6, IL-8, and TGFß but not TNFα.

Moreover, mesenchymal stem cells (MSCs) represent a transitional cell in the synovial tissue that is capable of differentiating into fibroblasts, maintaining their potential multilineage differentiation *in vitro*, with the main immunological function of controlling T-cell and B-cell activation and proliferation, blocking the activation of natural killer (NK) cells, reducing the antigen-presenting function of dendritic cells inducing the phenotypic transformation of macrophages, and reducing the apoptosis of polymorphonuclear (PMN) cells (13, 14). The anti-inflammatory functions of MSCs are exerted through the synthesis of IL-6, interleukin-10 (IL-10), TGFß, hepatocyte growth factor (HGF), and prostaglandin E2 (PgE2) (14). Of particular interest, the injection of human MSCs obtained from synovial tissue of patients with osteoarthritis (OA) into the joint cavity of collagen-induced arthritis (CIA) in DBA/1J mice model was associated with a decrease in TNF-α, IFN-γ, and interleukin-17A (IL-17A), while IL-10 production increased. Moreover, the number of T helper 1 (Th1) and Th17 cells in the spleen of mice treated with SM-MSCs (synovial membrane-MSCs) was decreased, while the number of Th2 (T helper 2), Treg (T regulatory), PD-1+ CXCR5+ FoxP3+ follicular Treg cells, and IL-10-regulated B cells was increased (15).

Finally, RA synovial tissues were characterized by the presence of nurse-like cells (NLCs), having the ability to promote antibody production by B cells, to protect lymphocytes from apoptosis, and to secrete a large number of cytokines and chemokines, i.e., monocyte chemotactic protein1 (MCP-1), IL-8, and other chemokines (16), as well as IL-6, IL-7, GM-CSF, and granulocyte colony-stimulating factor (G-CSF) (17), promoting the accumulation and activation of monocytes and lymphocytes, including B cells (18). RA-NLC has also the unique capacity to promote the differentiation of osteoclasts from myeloid precursors in a receptor activator of NF-κB/receptor activator of NF-κB ligand (RANKL) in an independent manner (14).

### IL-17 (and IL-8) as target cytokines in animal models of arthritis

Several studies have demonstrated that the synovial tissue of animal models of arthritis, such as CIA, adjuvant-induced arthritis (AA), antigen-induced arthritis (AIA), streptococcal cell wall (SCW) arthritis, and SKG (ZAP-70 mutation model, harbor a strain of a recessive mutation of the gene encoding an SH2 domain of ζ-associated protein 70 (ZAP-70), a key signaling molecule in T cells), express and synthesize an array of pro-inflammatory molecules (IL-1ß, IL-6, and TNFα) at the onset that determines the aggressive damaging disease phenotype (19). In particular, in the CIA model, the knock-out of the *IL-6* gene (*IL-6^−/−^*) led to complete protection from the onset of arthritis and a decrease of the anti-collagen type II autoantibody level (20). Interestingly, early neutralization of IL-6 bioactivities, after immunization with type II collagen, leads to a significant reduction of Th17 cells and subsequent protection from CIA (21). Mice knocked out for *IL-17* (IL-17^−/−^), 129/sc x C36BL/B6 F1 hybrid background, and IL-17^+/+^ littermates, showed CIA suppression, suggesting that IL-17 was crucial for collagen-specific T-cell activation and collagen-specific IgG2a synthesis (22). No data were available on the constitutive production of IL-17 by RA-FLS. This suggests that IL-6 plays an early effect in RA pathogenesis and is crucial to induce IL-17 synthesis. IL-17 is mainly (even if not exclusively) produced by specific T cells (Th17) that have been recruited into the synovial tissue, and which are generated under the influence of TGFβ, IL-6, and IL-1 (23), which are all molecules constitutively expressed by FLS. The key role of IL-17 in RA is supported by the observation that overexpression of IL-17 in DBA-1/BOM knee joint mice, as well as in SCW arthritis, induces joint inflammation, bone erosion, and cartilage proteoglycan loss (24). According to these data, IL-17 appears to amplify the inflammatory cascade triggered by IL-1α and β, TGFβ, and IL-6. Moreover, FLS cells constitutively express CXCL12 (stromal cell-derived factor 1, SDF-1) that recruits T cells which, in turn, constitutively express its receptor CXCR4 (25), and IL-6 that, through STAT3 activation, induces the expression of CCR3, CCR4, CCR5, and CXCR2 (26), all receptors present in CD3^pos^ cells associated with the synthesis of chemokines as CCL4 (macrophage inflammatory protein, MIP-1β), CCL5 (regulated upon activation, normally T cell expressed and secreted, RANTES), CCL17 (thymus and activation-regulated chemokine, TARC), and CXCL10 (interferon-y-inducible protein- IP-10) (25, 26). These chemokines contribute to the progressive accumulation of monocytes–macrophages infiltrating the tissue. In particular, RANTES, MIP-1β,α, and SDF-1 are more actively involved in the recruitment of T and B cells into the synovial pannus leading to the formation of extranodal lymphoid tissue and pseudo germinal centers, as seen in early and long-standing RA-derived synovial tissues (27). The massive recruitment of B and T lymphocytes cannot occur without neoangiogenesis, which is strongly favored by the chemokines that possess angiogenic properties, such as IL-8, CXCL12, CXCL5 (ENA-78, epithelial cell-derived neutrophil attractant 78), and CXCL1 (GRO alpha, growth-related oncogene alpha). Among the chemokines, IL8, which has functional homologs (CXCL1/KV, CXCL2/MIP-2, and CXCL5-6/LIX in rodents), plays a key role, since antagonizing its function by blocking one of its receptors (CXCR2) through an antagonist significantly reduced acute and chronic arthritis inflammation in rabbits (28). Indeed, the more inflammation in the tissue, the more inflamed the synovial fluid, which shows enrichment of infiltrating PMNs, strongly recruited by IL-8, CXCL5, and CXCL1. Therefore, the constitutive syntheses of IL-8 and SDF-1 by FLS strongly contribute to synovitis chronicity, allowing the continuous recruitment of PMNs into the joint cavity of T and B cells into the synovial tissue. In this scenario, IL-8 and CXCL12 are likely essential for the recruitment and maturation of Th17, and the constitutive production of IL-6 and IL-8 by FLS is crucial for the whole process of synovial tissue inflammation. Indeed, FLS cocultured with IL17 for 48 h, produced a high amount of IL-6 and IL-8 (29).

## Cellular crosstalk through IL-8 and IL-17

### IL-8

IL-8 is secreted by multiple cell types, including FLS, macrophages, monocytes, PMNs, endothelial cells, and MSCs (24), and its expression is regulated at both transcriptional and post-transcriptional levels (25). IL-8 mediates its effects *via* binding to two heterotrimeric G protein-coupled receptors, CXCR1 and CXCR2, that become phosphorylated, desensitized, and internalized. The signaling pathways activated downstream by CXCR1/2 engagement, including mitogen-activated protein kinase MAPK pathways (p38, MEK1/2, and JNK) (30). IL-8 signaling activates a wide range of transcription factors, such as nuclear factor kappa B (NFκB), activator protein-1 (AP-1), hypoxia-inducible factor 1 (HIF-1), and signal transducer and activator of transcription 3 (STAT3). It is chemotactic for PMNs, monocytes, and fibroblasts. Moreover, IL-8 also accelerates fibroblast migration and stimulates the deposition of tenascin and fibronectin during wound healing *in vivo*. In epithelial, endothelial, and fibroblastic cells, secretion of IL-8 is induced by IL-17 (31). It is one of the key chemokines regulating neoangiogenesis (28, 32). Importantly, IL-8 appears crucial in RA for the recruitment and infiltration of the synovium by leukocytes, and it is easily detectable in the synovial fluids and by *in situ* hybridization in the rheumatoid synovial tissue, correlating with disease activity (33, 34). In particular, IL-8 activates PMNs and subsequently releases neutrophil-derived chemotactic factor (NDCF) acting as chemotactic factors for monocytes and T cells (35). Inside the synovial tissue with lympho-myeloid pathotype, IL-8 is produced by CD20^pos^ germinal center (GC) lymphocytes, especially centroblasts and centrocytes, and it appears crucial, along with RANTES, to enhance the recruitment of T cells inside the GC and favor the interaction between B and T cells at a stage in which B lymphocytes are engaged in active-dependent interactions with T cells (36–38). In addition, IL-8 is produced by cells located within the *sublining layer* that is in direct contact with the cartilage, being one major adipokine secreted by stromal cells (39). If we consider that synovial stromal cells attract monocytes by producing IL-8 and MCP-1 (40) and IL-8 downregulates the expression of tissue inhibitors of metalloproteinases (TIMPs) (41), the increased synthesis of IL-8 in the sublining layer represents the ideal site for an increased expression of collagenases leading to cartilage damage (42). Moreover, IL-8 induces motility and loss of focal adhesion in primary fibroblasts and favors the direct invasion of cartilage by FLS and macrophages (37) that erode and degrade cartilage through the synthesis of collagenases (43–47). Since FLS cells constitutively express and synthesize IL-1β, and IL-1β is a strong inducer of IL-8 by chondrocytes (42), an amplification loop involving FLS and macrophages in the degradation and erosion of cartilage appears in place within the arthritis synovial tissue, with MMP14 (metallo type 1 matrix metalloproteinases) of critical importance (48–50).

In the joint “bare areas,” referring to the bone within the synovial space that is not covered by articular cartilage, the IL-8 recruitment of FLS and macrophages may lead to the key feature of inflammatory arthritis, that is, bone erosions (48, 49). In fact, IL-8 has been identified as an autocrine regulator of RANKL-induced osteoclastogenesis, by promoting the expression of RANKL by osteoblastic stromal cells (50, 51). Either osteoclast precursors or mature osteoclasts express CXCR1, the IL-8 receptor, and the IL-8 effect on osteoclasts and their progenitors is associated with CXCR1 cell surface expression, demonstrating a direct effect of IL-8 on osteoclast differentiation and activity (52). Moreover, the ablation of IL-8 with a neutralizing antibody, attenuated osteoclastogenesis by the inhibition of NFATc1 translocation to the nucleus, even in the presence of RANKL (53), clearly supporting the notion that IL-8 is an autocrine key regulator of osteoclasts differentiation and activation. This role of IL-8 may explain why 34.9% of patients with early RA (symptoms duration 6.4 ± 3.3 months) had erosions at diagnosis (49). The presence of Anti-citrullinated protein antibodies (ACPA), through the binding to citrullinated vimentin (as a putative autoantigen) present on the surface of osteoclasts, may increase cellular differentiation to bone-resorbing osteoclasts *via* autocrine stimulation of TNF production (44). In particular, ACPAs against vimentin or enolase, but not against other citrullinated peptides, were shown to induce the expression of IL-8 by osteoclast precursor cells, which acts in an autocrine way to facilitate osteoclastogenesis (54), thus stressing the role of the innate immune response in the erosion process, especially of seropositive arthritis. Since seronegative (for ACPA and rheumatoid factors) early RA also presents erosions in a significant percentage (31.9%) (55), the ACPA positivity does not appear to be a needful requisite for being erosive. In these cases, the IL-8-driven pathway may still play a key role.

### IL-17

There is no evidence of the constitutive IL-17 production by FLS in humans. In 1999, Chabaud et al. (56) demonstrated IL-17 in synovial biopsies of patients with RA. The IL-17 family is composed of six members (i.e., A–F) and is mainly produced not only by Th17 but also by CD8^+^ T cells, CD4-CD8-γδ T cells, NK cells, innate lymphoid cells (ILC3), mast cells (MCs), and PMNs, yet Th17 are thought to be the main drivers of arthritis (57, 58). Of interest, the MCs capture, store, and release bioactive IL17A (59, 60). In CIA models, Th17 and γδT cells were found in the synovial tissues, and Th17 depletion protected mice from bone erosions (61). In a seminal study, Komatsu et al. (62) showed that transferring CD25^hi^Foxp3^pos^CD4^pos^ or CD25^low^Foxp3^pos^CD4^pos^ T cells into mice immunized with type II collagen, and 1 week after secondary immunization, CD25^low^Foxp3^pos^CD4^pos^ T cells lost Foxp3 expression and became IL-17-producing cells. When culturing CD25^low^Foxp3^pos^CD4^pos^ T cells with Thy1^pos^CD11 cells (synovial fibroblasts) but not with Thy1^neg^CD11b^pos^ cells (synovial macrophages), Foxp3 was lost and became Th17-producing IL-17. The major mediator of the transition from a regulatory to active status of T cell was found to be IL-6, heavily produced by Thy1^pos^CD11b^neg^ cells (FLS), and the treatment with a neutralizing antibody against IL-6, while neither an antibody against TNF-α nor IL-1β, inhibited the generation of Th17 cells after the coculture of Foxp3^pos^CD4^pos^ T cells with synovial fibroblasts. These data suggest that IL-6, secreted by FLS, is crucial in the development of Th17 (along with IL1, TGFβ, and IL23) (62) and the production of IL-17. Furthermore, Th17 cells expressed high levels of RANKL and had higher osteoclastogenic ability than naive CD4^pos^ T cell-derived Th17 cells in a co-culture of synovial fibroblasts and bone marrow-derived monocyte and macrophage precursor cells (62). Moreover, IL-17 is able to enhance cartilage proteoglycan loss and inhibit its synthesis, as demonstrated in mouse models (56). On human RA-derived bone explants, IL-17 enhanced IL-6 production, increased bone resorption, and decreased its formation (56). Of critical importance, IL-17 induces the production of IL-6 and IL-8 from RA FLS *via* PI3Kinase/Akt-dependent pathways, thus further amplifying the inflammatory role of FLS in RA pathogenesis (63). In addition, the finding that IL-17 is directly involved in the stimulation of fibroblasts and endothelial cells to secrete cytokines such as IL-6, IL-8, G-CSF (granulocyte colony-stimulating factor), and prostaglandin E2, and in sustaining the proliferation of CD34^+^ hematopoietic progenitors suggests that this cytokine is not only an amplifier molecule but also a potent bridge between innate immune response and systemic inflammatory response (64). Finally, the demonstration that on one side IL-17 promotes B-cell chemotaxis (65)and on the other side that B cells stimulated with IL-4, IFNγ, IL-6, and TGFβ upregulate the expression of both IL-17A and F, appears to be an important step toward pannus expansion and maintenance (66).

In conclusion, IL-8 at the onset and IL-17 after the RA onset (58) are key cytokines involved in the amplification of the arthritis inflammatory process, with the expansion of FLS and the action on chondrolytic and osteolytic activation.

## IL-8 and IL-17 as key players in organ damage (cardiovascular risk, lung interstitial fibrosis, chronic obstructive pulmonary disease, and chronic pain) in RA.

### IL-8

The expression of IL-8 is induced not only by several inflammatory cytokines but also by oxidized low-density lipoprotein (OxLDL) that mediates cholesterol loading of macrophages, which, in turn, selectively reduces the production of tissue inhibitors of metalloproteinases-1 (TIMP-1) by human monocyte-derived macrophages (HMDMs) (40). The role in cardiovascular disease is supported by the demonstration that myocardial ischemia and reperfusion strongly increase IL-8 mRNA expression, which determines leukostasis and PMNs-mediated myocardial injury (67), and by the observation that inhibiting IL-8 with a monoclonal antibody protects the myocardium from the injury of ischemia–reperfusion, as seen in a New Zealand White (NZW) mouse model (68). Moreover, the demonstration that in interstitial lung disease-idiopathic pulmonary fibrosis (ILD-IPF), IL-8 and its receptor CXCR1 are highly expressed by mesenchymal progenitor cells (MPCs), which are the cellular originators of IPF fibroblasts, is of clinical significance. Most importantly, IL-8 was shown to expand the MPCs population, recruit activated macrophages, and promote fibrosis (69).

Finally, musculoskeletal chronic inflammatory diseases are often associated with acute and chronic pain. The demonstration that IL-8 plays an important role in neuropathic pain (following nerve injury) (70), and the observation that an inhibitor of IL-8-CXCR1/2 interaction improved neuropathic pain (71), suggest that IL-8 may indeed play a role in chronic neuropathic pain that affects 17–21% of patients with RA (72).

### IL-17 family

Within its biological effects, IL-17A might promote accelerated atherosclerosis, which is a key extra-articular manifestation of RA (73). The observation that in the ApoE^−/−^ (apolipoprotein E-deficient) mouse model, which is a model prone to accelerated atherosclerosis, IL-17A blockage reduced the plaque burden, IL-6 levels, G-CSF levels, CXCL1 expression, and macrophage content in the aorta strongly suggests, among many others, a pathogenetic role of IL-17A in the atherogenic process (74). The clinical observation that RA patients who had higher baseline bioactive peripheral blood IL-17 levels and were followed for 19.8 years had a higher incidence of stroke, acute myocardial infarction (AMI), or peripheral acute artery ischemia, supports the reported experimental data (75).

Considering the lung involvement in RA, interesting data have emerged from studies on mice models deficient in the IL-17 receptor A (IL-17Ra^−/−^), showing that in two different mice models of pulmonary fibrosis employing COPD stimuli (cigarette smoking and viral mimetic polyinosinic-polycytidylic acid), animals were protected from both airway inflammation and fibrosis (76). Moreover, blocking IL-17A or IL-17Ra with monoclonal antibodies proved that IL-17A exerts crucial functions in this process. In fact, the IL-17A/IL-17Ra axis has been demonstrated to be critical in murine fibrosis models (77), and most importantly, it was demonstrated, *in vitro*, that IL-17A-stimulated human fibroblasts proliferation can be suppressed by inhibitors of Janus Kinase 2 (JAK2) or JAK1-3, while myofibroblast transdifferentiation is sensitive to JAK2 inhibition (78).

**Figure 1:**
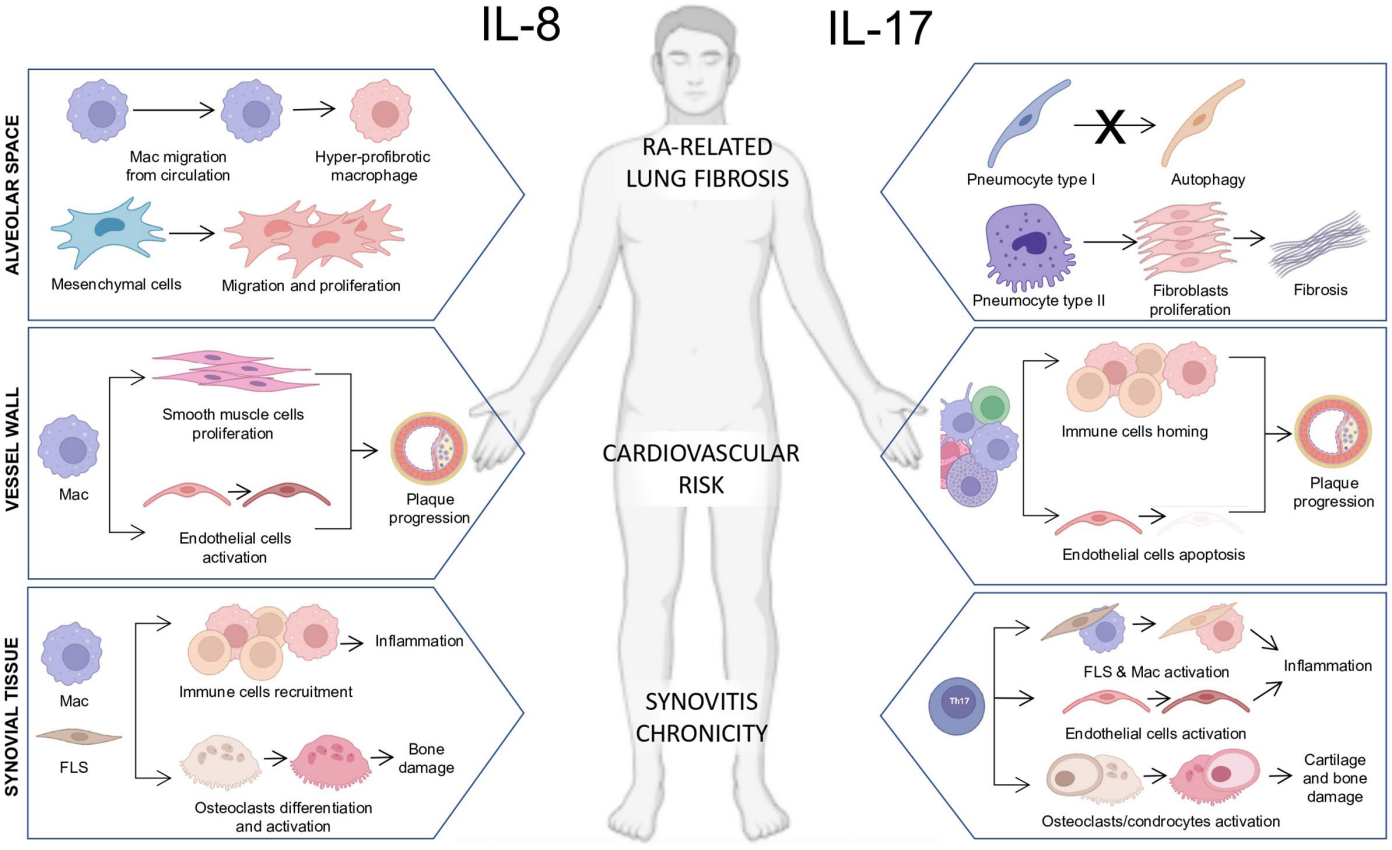
Pathogenetic actions of interleukin-8 (IL-8) and IL-17 on immune and stromal cells in multiple biological compartments in rheumatoid arthritis. (Left section) Schematic representation of the pathogenetic actions of IL-8 in the synovial tissue, blood vessel wall, and alveolar space in RA. *Synovial tissue*: The release of IL-8 by resident macrophages and fibroblast-like synoviocytes (FLS) plays a chemotactic action promoting the recruitment of immune cells toward the synovial tissue fueling tissue inflammation and promoting osteoclasts differentiation and activation leading to bone damage. *Vessel wall*: The release of IL-8 by infiltrating macrophages promotes the proliferation of smooth muscle cells and the activation of endothelial cells promoting plaque progression. *Alveolar space*: The release of IL-8 by alveolar macrophages enhances myeloid cell migration from circulation and promotes the development of hyper-fibrotic macrophages. Moreover, the release of IL-8 from mesenchymal cells induces their migration and proliferation in an autocrine loop. (Right section) Schematic representation of the pathogenetic actions of IL-17 in the synovial tissue, blood vessel wall, and alveolar space in RA. *Synovial tissue*: The release of IL-17 by Th17 lymphocytes promotes the activation of synovial macrophages and FLS contributing to the chronicity of synovitis. Moreover, IL-17 is a potent inducer of osteoclast/chondrocyte activation contributing to bone and cartilage damage. *Vessel wall*: The release of IL-17 by adaptive and innate immune cells promotes the chemotaxis of immune cells within the vessel plaque and promotes endothelial cell apoptosis, accelerating the atherosclerotic process and leading to increased cardiovascular risk. *Alveolar space*: The release of IL-17 interferes with the pneumocyte I autophagy process and its release by pneumocytes type II enhances fibroblast proliferation, which contributes to extracellular matrix deposition and lung fibrosis.

The IL-17/IL-17R axis has a role even in chronic pain in RA, as emerged from experimental data in a mouse model of neuropathic pain elicited by partial ligation of the sciatic nerve, in which the IL-17 knock-out mice demonstrated significantly lower pain hypersensitivity, decreased infiltration of T cells and macrophages into the sciatic nerve, and decreased activation of microglia and astrocytes in the L3–L5 dorsal and ventral horns of the spinal cord. Conversely, IL-17 infusion increased mechanical allodynia and thermal hyperalgesia (79). This study demonstrates that, in experimental models, IL-17 increases glial activation and neuropathic pain after peripheral nerve injury, and if we consider that IL-17A crosses the brain barrier to trigger neuroinflammation in rat models (80), we may infer that IL-17A indeed is a player in one of the most complex symptoms of pathophysiology, the occurrence of neuropathic pain in chronic inflammatory illnesses (Figure 1).

## IL-8 and IL-17 and monoclonal antibodies

Experimental data and synovial tissue analyses have demonstrated the presence and function of IL-8 and IL-17 in RA. Kraan et al. showed a higher expression of IL-8/CXCL8 in the involved joints of patients with RA compared with uninvolved ones (35), and Koch et al. demonstrated the crucial pro-angiogenetic role of IL-8 in the development of chronic synovitis in RA (81). When examining peripheral blood and synovial fluid compartments, IL-8 levels were significantly increased in the plasma of very early RA (VERA) compared with established RA or healthy controls and in the synovial fluid of established RA compared with osteoarthritis patients (82). Despite this, the results obtained from the use of anti-IL-8/CXCL8 in a clinical trial enrolling patients with RA have not been published, and the compound was not further developed (83).

Moreover, synovial tissue of patients with RA is significantly enriched in IL-17 (84, 85), and IL-17 receptors were identified in the endothelial cells of RA synovium and chondrocytes derived from many types of arthritis, with the highest expression in osteoarthritis and SpA cartilages and the lowest in RA cartilage (82). Similar to IL-8, the peripheral blood of patients with VERA is enriched in IL-17 compared with established RA or healthy controls, and the synovial fluid of patients with established RA shows significantly higher IL-17 levels compared with osteoarthritis (82).

However, despite a strong biological background, the efficacy of secukinumab (a fully human monoclonal antibody binding and neutralizing selectively IL-17A) in RA was modest when compared with abatacept (86), with limited efficacy in patients who were poor responders to TNF inhibitors (87). We know that chronically stimulated lymphocytes undergo TCR-zeta downregulation and become TCR-zeta^dim^ lymphocytes. TCR-zeta^dim^ lymphocytes are increased in RA synovial tissue and would selectively migrate to the joint during disease flare. We, and others, found that TCR-zeta^dim^ lymphocytes not only produce IL-17 but also IFNγ (88). These T cells are called non-classical Th1 because they produce IFNγ similar to Th1, yet they have been shown to proliferate more than Th17 after stimulation and to produce an increased amount of pro-inflammatory cytokines (GM-CSF, TNFα, IL2, IL17, and IFNγ), and might be more resistant to IL-17-targeted therapy (89). Tenosynovitis occurs in a subset of patients with RA, even in patients with no metacarpophalangeal joint synovitis (90), and these patients could benefit from an anti-IL-17A therapeutic approach if they do not fully respond to the specific biologic agent (83). Although no data are available on patients’ refractory to anti-TNF, a bifunctional antibody might be relevant, as observed in other illnesses (91).

### How can we support the IL-8 and IL-17 involvement in RA?

The answer comes from the biology of RA. Studying the effects of gold sodium thiomalate (GST) and methotrexate (MTX) in RA, both employed as first-line therapy in RA diagnosis, Seitz et al. (92) showed that after stimulation with lipopolysaccharide (LPS) or IL-1ß, there was a profound decrease of IL-8 levels in peripheral blood mononuclear cell (PBMC) cultures from healthy controls as well as patients with RA, and also a significant decrease in the spontaneous production of IL-8 by RA PBMCs. If we consider that IL-8 is released by several cells which populate the synovial tissue of RA (93, 94) (endothelial cells, FLS, MLS, and chondrocytes), we may hypothesize that control of the innate immune response by MTX may be obtained through the inhibition of IL-8 synthesis. The demonstration that tocilizumab (a monoclonal antibody directed against the IL-6 receptor) was able to inhibit the synthesis of IL-8 from triple-negative breast cancer cells, thus blocking both IL-6 and IL-8, which promotes angiogenesis and favor the growth and spread of the disease, suggests that targeting IL-6 means targeting also IL-8, with IL-6 coming first (95). Since MTX also decreased IL-6 synthesis after stimulation with LPS of PBMC from juvenile idiopathic arthritis (JIA) (96) and in the GPI-arthritis model (glucose-6-phosphate isomerase-induced arthritis model), MTX potently inhibits the development of arthritis, and this effect relates to the progressive reduced SLC19A1 expression (the folate carrier SCL19A1) (97). Thus, it is possible to infer that the inhibition of IL-6 is a key to downregulating IL-8. In contrast, when studying gene expression in whole blood-derived cells, patients with RA stabilized under etanercept (a fusion protein produced by recombinant DNA that fuses the TNF receptor to the constant end of the IgG1 antibody) showed an increased expression of the IL-8 gene, as well as under infliximab therapy (a DNA-derived chimeric monoclonal antibody working by binding to and neutralizing TNFα) (98). The induction of a strong pro-inflammatory gene such as IL-8, under TNFα inhibition, suggests that an underlying persistent inflammatory milieu may be associated with RA clinical stabilization. Whether this may foresee the progressive loss of efficacy, needs to be defined in follow-up studies of patients treated with TNF inhibitors. In contrast, Lun et al. (99) showed a significant decrease in TNFα and IL-8 released by peripheral blood-derived mononuclear cells stimulated with phytohaemagglutinin and LPS under infliximab treatment, without any change in the induction of IL-6, IL-1ß, and IL-18. These apparently contradictory results support the idea that a persistent inflammatory background may remain after TNFα inhibition.

If IL-6 is upstream in RA disease, it follows that targeting IL-6 means also targeting IL-8, while a direct inhibition of IL-8 may not stop the whole inflammatory cascade. Further support to the rationale that IL-6 and IL-8 walk together is the demonstration that RA-derived FLS stimulated with TNFα and IL-17 showed a higher synthesis of IL-6, IL-8, and G-CSF. Much lower concentrations of TNFα than IL-17 were required to stimulate chemokines, with IL-6 stimulated at higher amounts than IL-8. Interestingly, a bispecific antibody against TNF and IL-17 blocked their expression and synthesis much more than it can be done using single selective inhibitors. Consequently, the anti-arthritic effect of the bispecific antibody became evident in the TNFα human transgenic arthritis model — hTNF^tg^ (C57Bl6 background, Tg197 strain) (100).

If targeting IL-6 leads to an increase in the Treg/Th17 ratio, since the conversion of Treg cells (Foxp3 positive) into Th17 cells is IL-6 dependent (62), a tocilizumab treatment increased this ratio *in vivo* (101). Once again, targeting IL-6 means targeting IL-17 downstream, and since TNFα induces IL-6 and IL-8, targeting IL-6 means also stopping the deleterious effects of TNFα. This can be the reason why IL-6 has been considered the pivotal cytokine in RA (102). Indirect supports to these considerations come from recent data showing that abatacept (a fusion protein composed of the Fc region of the immunoglobulin IgG1 fused to the extracellular domain of CTLA-4, an immune check-point that downregulates T-cell activation and the immune response) leads its strongest effects in patients with the lowest IL-6 levels at the baseline and with the highest Treg levels (103).

### Unmet needs and perspectives

Despite incredible advances in our understanding of the cellular and molecular biology of RA, an important percentage of patients cannot be led to full persistent remission. We recognize that IL-6 (upstream pivot) and TNFα (final kingmaker) represent key players in treatment options, yet there are patients who we define as difficult to treat (104) that still do not reach a satisfactory clinical control with the available therapies (Figure 1). To avoid multiple interventions with multiple drugs with different modes of action, following the pathogenetic pathways described here, a sequential therapy like in hematology (105), with anti–IL-6 or anti-TNFα followed by anti–IL-17, might offer favorable outcomes. In the CIA model, the dual blockade of TNF and IL-17 gave better results than either monotherapy (106) and in the TNFα transgenic animal model of arthritis, as well as cartilage and bone damage, were better controlled with bi-specific antibodies (anti-TNFα and anti–IL-17A) than with monotherapies (100). Along the same line, in the CIA model, the therapeutic intervention during the induction phase of arthritis with anti–IL-6 and anti–IL-21 reduced disease development more efficaciously than monotherapies (107).

It is well accepted that patients with RA are clinically heterogeneous and that the lympho-myeloid pathotype at synovial tissue analysis represents the most aggressive and severe subset (108). In this scenario, RA patients with resistant tenosynovitis might benefit from anti–IL-6, which has been shown to also act on the stromal component (108), followed by anti–IL-17 therapy (106). Conversely, in RA patients with an erosive disease, anti–IL-6 or anti-TNFα, followed by anti–IL-8, which is a key factor promoting erosiveness, should be tested. Alternatively to anti–IL-8 inhibitors, not currently available in the clinic, JAK inhibitors could be employed sequentially (109, 110), some of which (i.e., peficitinib and filgotinib) may act on FLS reducing their migration invasiveness and decreasing their synthesis of IL-8 and IL-6 (111). All of these unmet needs require intensive and tireless biopsy-driven research at the cellular (112) and molecular levels (113), aimed to develop a more biologically oriented personalized decisional algorithm. While waiting for bifunctional antibodies, the dual-target with anti-TNFα (certolizumab), followed by targeting IL-17A-F (ixekizumab), led to faster and stronger disease control in anti-TNFα incomplete responders, at the expense of more, not severe, infections (114).

Recent studies on the genetic background of seropositive (rheumatoid factor and anti-citrullinated protein antibodies positive) RA suggest that the inflammatory milieu is very complex (113). The association between seropositive RA and STAT4 (significantly associated with RA and activated by a variety of cytokines, i.e., IL-12, type 1 IFN, IL-23, IL-2, IL-27, and IL-35), PTPN22 (important for TCR and BCR signaling, for the upregulation of IL-4R, IL-13R, IL-17R, and IL-21R, the hyper citrullination, and netosis) (112), FLT3 (important for the maintenance of hematopoietic progenitors, development of B cell lineage, and dendritic cells), gene variants, CTLA4, and IL6ST, supports the rationale that along with the major final players, other molecules are certainly involved in different phases.

## Conclusion

The availability of synovial tissue biopsy during the RA course might provide information on the aggressiveness of synovitis (19) in terms of stromal cells proliferation and enrichment of B-cell lineage (105) and can be integrated with the assessment of the activity across the disease stages through multiparametric activity scores (106) to create patient-specific individual taxonomy. This will allow clinicians to define much earlier the individual molecular signature and the entity of the response to therapy and will let them promptly modify the therapeutic approach. Therefore, an oncohematological-like approach with drugs modulating different targets could be achievable once the clinical features and tissue characteristics have defined this individual multiparametric matrix.

## Author contributions

EG, GF, and SA conceived the study. EG, BT, DB, SP, and SA collected the literature data. BT, DB, SP, and SA drafted the figure. EG, BT, DB, SP, BT, SA, and GF drafted and revised the manuscript. All authors contributed to the article and approved the submitted version.

## Conflict of interest

The authors declare that the research was conducted in the absence of any commercial or financial relationships that could be construed as a potential conflict of interest.

## Publisher’s note

All claims expressed in this article are solely those of the authors and do not necessarily represent those of their affiliated organizations, or those of the publisher, the editors and the reviewers. Any product that may be evaluated in this article, or claim that may be made by its manufacturer, is not guaranteed or endorsed by the publisher.
